# 3D Variation in delineation of head and neck organs at risk

**DOI:** 10.1186/1748-717X-7-32

**Published:** 2012-03-13

**Authors:** Charlotte L Brouwer, Roel JHM Steenbakkers, Edwin van den Heuvel, Joop C Duppen, Arash Navran, Henk P Bijl, Olga Chouvalova, Fred R Burlage, Harm Meertens, Johannes A Langendijk, Aart A van 't Veld

**Affiliations:** 1 Department of Radiation Oncology, University of Groningen, University Medical Center Groningen, Groningen, the Netherlands; 2 Department of Epidemiology, University of Groningen, University Medical Center Groningen, Groningen, the Netherlands; 3 Department of Radiation Oncology, The Netherlands Cancer Institute, Antoni van Leeuwenhoek Hospital, Amsterdam, the Netherlands

**Keywords:** Interobserver variability, Interobserver agreement, Head and neck cancer, Organs at risk, Delineation

## Abstract

**Background:**

Consistent delineation of patient anatomy becomes increasingly important with the growing use of highly conformal and adaptive radiotherapy techniques. This study investigates the magnitude and 3D localization of interobserver variability of organs at risk (OARs) in the head and neck area with application of delineation guidelines, to establish measures to reduce current redundant variability in delineation practice.

**Methods:**

Interobserver variability among five experienced radiation oncologists was studied in a set of 12 head and neck patient CT scans for the spinal cord, parotid and submandibular glands, thyroid cartilage, and glottic larynx. For all OARs, three endpoints were calculated: the Intraclass Correlation Coefficient (ICC), the Concordance Index (CI) and a 3D measure of variation (3D SD).

**Results:**

All endpoints showed largest interobserver variability for the glottic larynx (ICC = 0.27, mean CI = 0.37 and 3D SD = 3.9 mm). Better agreement in delineations was observed for the other OARs (range, ICC = 0.32-0.83, mean CI = 0.64-0.71 and 3D SD = 0.9-2.6 mm). Cranial, caudal, and medial regions of the OARs showed largest variations. All endpoints provided support for improvement of delineation practice.

**Conclusions:**

Variation in delineation is traced to several regional causes. Measures to reduce this variation can be: (1) guideline development, (2) joint delineation review sessions and (3) application of multimodality imaging. Improvement of delineation practice is needed to standardize patient treatments.

## Background

Radiotherapy (RT) plays an important role in the treatment of head and neck cancer patients. Many new radiation delivery techniques such as intensity-modulated RT (IMRT) have been developed to allow improved dose conformation with steeper dose gradients compared with conventional three-dimensional conformal RT. Variation in contouring is an important obstacle in the development of high geometric accuracy in the clinical application of these new techniques. Reproducibility in delineation of tumour and normal structures is of importance for optimal patient treatment [1]. As new radiation delivery techniques are increasingly controlled by OAR constraints for normal tissue sparing [2], variations in OAR delineation may unintentionally influence the treatment plan including the dose to these OARs [3]. In a number of publications (e.g. Bortfeld and Jeraj [4]), uncertainties in the contouring of organs is also mentioned as one of the potential causes for uncertainties in historical dose and volume data and therefore reduced performance of predictive models. Deasy et al. [5] furthermore mentioned that differences in segmentation procedure could be one of the reasons explaining variations between existing models.

Target volume delineation variability in the head and neck area has been investigated in several studies (e.g. Rasch et al. [6]), indicating the need to minimize observer variation for adequate irradiation. However, interobserver variability of OARs in the head and neck area has not been frequently studied. Nelms et al. [3] found significant organ-specific interclinician variation for head and neck OARs. These variations resulted in large differences in dose distribution parameters, especially in high dose gradient regions. The authors stated that the major variations were in each observer's interpretation of the OARs actual size and shape, suggesting the need for basic training (with unambiguous guidelines) on identifying OARs. Our department uses well-defined delineation guidelines to promote the consistency and accuracy of delineation such as the recently described guidelines for the delineation of OARs related to salivary dysfunction and anatomical structures involved in swallowing [7,8]. Interobserver variation in the contouring of OARs is therefore intended to be minimal, but still there will be regions in the OARs which are difficult to interpret for the observer. Accurate determination of variation in OAR delineation expressed in volumetric, positional and local 3D measures is therefore needed to establish current accuracy status, to bring actual weaknesses to light and to establish measures to reduce current redundant variability in delineation practice. More consistency in the delineation of OARs may contribute to more consistent dose volume data, and thus less uncertainty in the usage of dose volume characteristics. With the unambiguous and consistent contouring of OARs we could furthermore generalize the application of normal tissue complication probability (NTCP) models. We might even be able to develop improved models, when more consistent dose-volume data is correlated with clinical outcome. This is particularly important for the rising application of particle therapy, in which the dose gradients are extremely steep. The obtainable level in accuracy of delineations also provides valuable information for the evaluation of tools for automatic (re-)contouring. Qazi et al. [9], for instance, reported high accuracy for automatic segmentation within a clinically-acceptable segmentation time, but also mentioned the need for multi-observer studies to give more insight in the robustness, reliability, and stability of the automated approach. Existing variations in expert delineations could serve as benchmark data.

The aim of the current study was to indicate OAR regions with high interobserver variability in the head and neck area, to subsequently establish possible solutions for this variability in delineation practice.

## Methods

### Patients

The study population was composed of 6 head and neck cancer patients. These patients underwent a planning CT scan (CT_plan_) which was acquired prior to radiation, and a repeat CT scan (CT_rep_) which was acquired during the course of radiation. CT_rep _scans were performed 11 to 35 days (range) after the start of radiotherapy. The CT images were made with the patient in supine position on a multidetector-row spiral CT scanner (Somatom Sensation Open, 24 slice configuration; Siemens Medical Solutions, Erlangen, Germany). The acquisition parameters were: gantry un-angled, spiral mode, rotation time 0.5 s, 24 detector rows at 1.2 mm intervals, table speed 18.7 mm/rotation, reconstruction interval 2 mm at Kernel B30 and 120 kVp/195 mA. The matrix size was 512 × 512, with a pixel spacing of 0.97 × 0.97 × 2.0 mm in the x, y and z directions, respectively.

Five specialized head and neck radiation oncologists (R.S., A.N., H.B., O.C. and F.B.), all treating more than 50 head and neck patients per year, delineated five OARs on axial CT slices in all CT images. The radiation oncologist did not have clinical patient information additional to the CT scan. The OAR set included the spinal cord, the parotid and submandibular glands, the thyroid cartilage, and the glottic larynx. For one patient, the right parotid gland contained tumour infiltration and therefore the patient was excluded from analysis for this particular OAR beforehand. The total number of delineated structures was 410.

CT_plan _and CT_rep _were delineated under slightly different circumstances, since CT_plan _was made with contrast-enhancement (iodine containing contrast medium, intravenously applied) while CT_rep _was acquired without contrast enhancement. Furthermore, the CT_plan _scan was delineated from scratch and the CT_rep _scan was delineated using a template obtained from the delineated contours of the CT_plan_, which were propagated to CT_rep _after a rigid registration of CT_rep _to CT_plan _in each individual patient.

### Delineation guidelines

The radiation oncologists were instructed to delineate the parotid and submandibular glands according to the delineation guidelines of van de Water et al. [7].

Following these guidelines the parotid gland was demarcated in lateral direction by a hypodense area corresponding to subcutaneous fat and more caudally by the platysma. The medial border was defined by the posterior belly of the digastric muscle, the styloid process and the parapharyngeal space. The cranial aspect of the parotid gland was related to the external auditory canal and mastoid process. Caudally, the gland protruded into the posterior submandibular space inferior to the mandibular angle. The anterior border was defined by the masseter muscle, the posterior border of the mandibular bone and the medial and lateral part of the pterygoid muscle. The posterior border was delimited by the anterior belly of the sternocleidomastoid muscle and the lateral side of the posterior belly of the digastric muscle. The external carotid artery, the retromandibular vein and the extracranial facial nerve are prescribed to be enclosed in the parotid gland.

Cranial demarcation of the submandibular gland was defined by the medial pterygoid muscle and the mylohyoid muscle, the caudal demarcation by fatty tissue. The anterior border was the lateral surface of the mylohyoid muscle and the hyoglossus muscle, and the posterior border the parapharyngeal space and the sternocleidomastoid muscle. Lateral demarcation was described by the medial surface of the medial pterygoid muscle, the medial surface of the mandibular bone and the platysma. The medial border was finally described by the lateral surface of the mylohydoid muscle, the hyoglossus muscle, the superior and middle pharyngeal constrictor muscle and the anterior belly of the digastric muscle.

The spinal cord was delineated as the actual spinal cord instead of using bony structures as surrogate for the spinal cord, starting at the tip of the dens and ending at the level of the third thoracic vertebra. The thyroid cartilage was delineated as the actual thyroid cartilage. The cranial border of the glottic larynx was defined as the arythenoid cartilages and the caudal border as the edge of the cricoid.

### Statistical considerations

For all observers, mean volumes and standard errors (SEs), as well as coefficients of variation (CVs) per OAR were calculated. In addition, an 'OAR ratio' for each observer was determined, which was defined as the ratio of the mean OAR volume per observer divided by the mean volume of that OAR determined by all observers. Friedman's test was applied to the CT_plan _data per OAR separately to investigate a possible systematic effect in the determination of volumes by the observers.

We used different endpoints to investigate interobserver variability. Variations in volume were indicated by the Intraclass Correlation Coefficient (ICC), and differences in combined volume and positional variations by the Concordance Index (CI). Local variations in delineation were finally described by the regional 3D SD. Integration of these three endpoints could help us to identify the type of variation in delineation.

### Intraclass correlation coefficient

Analysis of variance (ANOVA) was conducted for estimation of the ICC per OAR. The ICC quantifies how well the observers defined the same size of volumes, without considering the position of the volume of one observer with respect to the other [10]. To assess the ICC for each OAR separately, a three-way mixed effect analysis of variance model was applied to the volume data. All possible interaction terms were included, with patients and observers as random effects and time as a fixed effect. The time effect describes the mean difference in volume during the treatment (CT_plan _vs. CT_rep_). The patient and time-patient interaction effects were considered sources of variation that are unrelated to observer variation. Therefore, in line with Barnhart et al. [11], the ICC was calculated as the ratio of the sum of variance components for patient and time-patient interaction effects and the sum of all variance components. It represents the correlation coefficient of two arbitrary observers measuring the same patient at the same time (the same CT scan). We used a classification of the data as presented by Shrout et al. [12]. Values of 0.00-0.10 represent virtually no agreement (reliability); 0.11-0.40 slight agreement; 0.41-0.60 fair agreement; 0.61-0.80 moderate agreement; and 0.81-1.00 substantial agreement.

### Concordance index

Another endpoint for interobserver variability used in this study was the ratio of the intersection (Volume1∩Volume2) and union (Volume1∪Volume2) volume of two delineated volumes. Terminology for this coefficient varies [13] but we adhered to the term concordance index (CI), as is also done in the overview of Hanna et al. [14] and in the review of Jameson et al. [15]. The CI is both sensitive to positional differences and differences in volume size between observers.

We calculated a mean CI value per OAR by averaging all individual CIs over all ten observer pairs and all twelve CT scans, and we determined the range of CIs. A CI of 1.00 indicates perfect overlap (identical structures), whereas a CI of 0.00 indicates no overlap at all.

Large discrepancies between the ICC and the CI indicate that observers are either more consistent in defining the volume size (ICC > > CI), or more consistent in positioning the volumes (CI > > ICC).

### 3D analysis of variation

The 3D analysis of variation allows quantification of local variation in delineated structures in 3D [16]. For each OAR, a median contour surface of all 5 observer delineations was computed in 3D [16,17]. The local variation in the five distances to the median (SD) was determined for each surface point, and was averaged over all surface points of the OAR to obtain the *global *3D SD.

**Figure 1:**
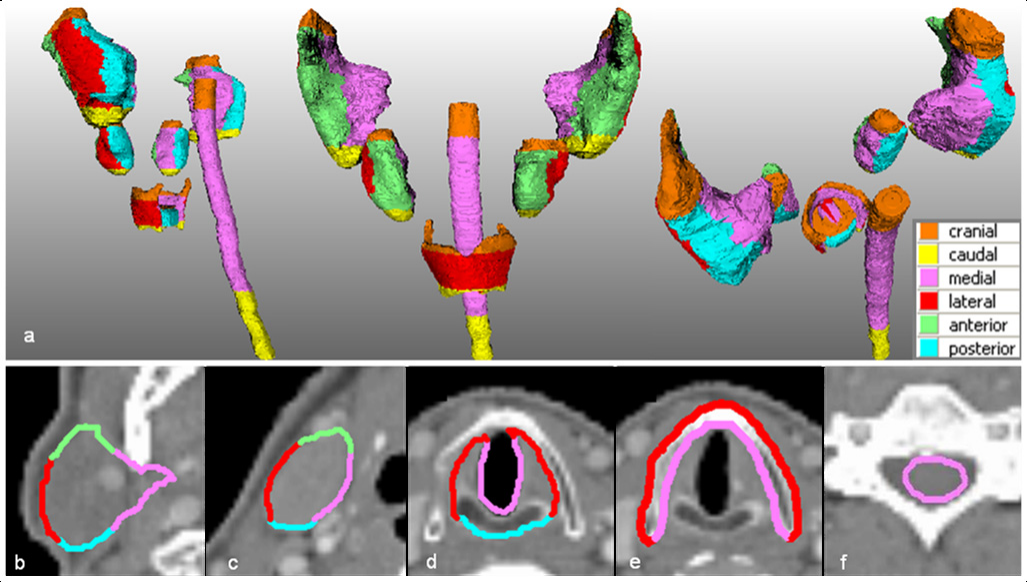
**Division of the studied OARs in sub regions for studying the regional 3D variations in delineation**. (**a**) Left side- (left), frontal (middle) and rear (right) 3D view of the studied organs at risk (OARs); the parotid and submandibular glands, spinal cord, thyroid cartilage and glottic larynx, divided in sub regions according to the colour legend. (**b**-**f**) Transverse central slices of the parotid gland (**b**), submandibular gland (**c**), glottic larynx (**d**), thyroid cartilage (**e**) and spinal cord (**f**) showing the division in sub regions according to the colour legend.

For further analysis, the OARs were divided into several anatomical sub regions. For the parotid glands, the upper and lower 5 slices were assigned as the cranial and caudal sub region, for the submandibular glands the upper and lower 3 slices were used. The anterior and posterior sub regions of the parotid gland were defined to be lateral to the mandibular bone. The spinal cord was defined in a cranial (up to C1), medial (C2 to T1) and caudal (from T2 on) sub region. The cranial and caudal sub regions of the glottic larynx were defined to be the upper and lower slice of the contour, respectively. The cranial sub region of the thyroid ended at the point where the median contour consisted of a closed structure in the transverse view. The caudal unclosed part of the contour was defined to be the caudal sub region. Figure 1 shows a 3D representation of the sub regions (a), together with transverse central slices of the OARs (b-f) to illustrate the anterior, posterior, medial and lateral sub regions. Calculation of the SD of a particular sub region resulted in a *regional *3D SD.

Note that these 3D SD results provided additional information to the ICC and CI, because these results quantify in which region of the OAR the highest variability in volume sizes and position was observed.

## Results

### Descriptive statistics

**Figure 2:**
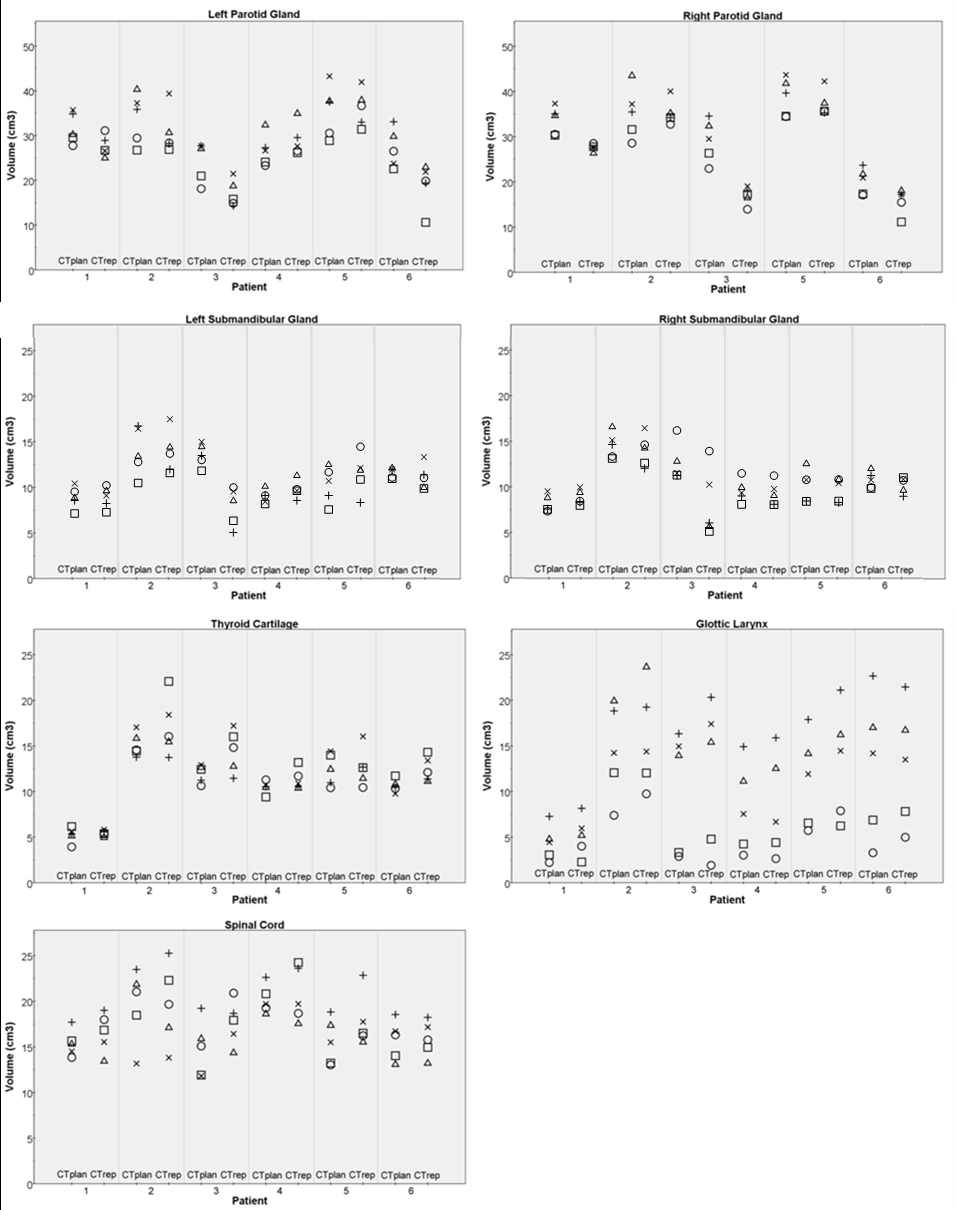
**Variation in the definition of organ at risk volume**. Volumes of the organs at risk according to observer 1(○), 2(□), 3(×), 4(Δ) and 5 (+). CT_plan _is planning CT and CT_rep _is repeat CT scan. The right parotid gland data contains 5 patients instead of 6 because of the exclusion of one gland (of patient 4) due to tumour infiltration.

Table 1 and Figure 2 present an overview of the volume data. Variation between observers in individual patients as well as between patients was seen. Planning and repeat CT sets are depicted separately, but no general trend in the differences between both CT scans was observed. For each OAR a certain systematic observer effect in the determination of the volumes was seen. Observer 1 and 2 defined significantly smaller volumes for the parotid and submandibular glands than the other observers (Friedman Test, *p *< 0.005). For the glottic larynx and spinal cord observer 1 seem to define the smallest volumes while observer 5 defines the largest volumes (Friedman Test, *p *< 0.006). These results were in line with the mean OAR ratios (Table 1).

**Table 1:** Mean volume, coefficient of variance, and mean OAR ratio per observer and organ at risk

OAR	Mean volume	CV	Mean OAR ratio*				
			
	[cm^3^] (SE)		obs. 1	obs. 2	obs. 3	obs. 4	obs. 5
Spinal cord	17.6 (1.1)	16%	0.98	0.98	0.91	0.92	1.17

Parotid gland left	28.4 (2.6)	15%	0.92	0.85	1.10	1.08	1.03

Parotid gland right	29.6 (3.9)	12%	0.88	0.90	1.06	1.04	1.02

Submandibular gland left	10.6 (0.9)	16%	1.06	0.86	1.12	1.06	0.95

Submandibular gland right	10.4 (0.9)	16%	1.09	0.88	1.06	1.03	0.90

Thyroid cartilage	11.4 (1.5)	14%	0.96	1.11	1.11	0.97	0.93

Glottic larynx	10.3 (2.8)	56%	0.45	0.60	1.13	1.38	1.66

OAR = organ at risk, SE = standard error of the mean, CV = coefficient of variance, obs. = observer

SE and CV were determined by the results of ANOVA, the CV represents variability due to observers

*OAR ratio = the volume of the OAR as determined by each observer divided by the mean volume of all five observers

The CVs in Table 1, which indicate an observer relative standard deviation for observing a volume, clearly showed highest variability for the glottic larynx (56%), while the other CVs varied from 12 to 16%.

### Intraclass correlation coefficient

The ICCs (Table 2) indicated largest observer variation (lowest ICC) in the volumes of the glottic larynx (ICC = 0.27) and the spinal cord (ICC = 0.32). Both OARs were classified to have slight agreement for delineation of volume sizes. The submandibular glands showed fair to moderate agreement (ICC = 0.60 and 0.61) for consistent volume delineation while the parotid glands (ICC = 0.65 and 0.86) and the thyroid cartilage (ICC = 0.83) showed moderate to substantial agreement.

**Table 2:** Interobserver variability of the organs at risk described by 3 different endpoints

OAR	ICC	CI (min-max)	3D SD (mm)						
			**Global**	**Region**					
				
				**cranial**	**caudal**	**medial**	**lateral**	**anterior**	**posterior**

Spinal cord	0.32	0.64 (0.41-0.83)	1.5	2.1	2.4	0.9	-	-	-

Parotid gland left	0.65	0.69 (0.43-0.83)	2.6	3.3	2.8	2.8	1.2	2.0	1.9

Parotid gland right	0.86	0.71 (0.50-0.86)	2.0	2.8	2.4	2.4	0.9	1.5	1.5

Submandibular gland left	0.61	0.70 (0.46-0.85)	1.8	2.9	0.7	1.7	1.7	1.6	1.4

Submandibular gland right	0.60	0.71 (0.36-0.83)	1.5	2.7	0.8	1.4	1.1	1.4	1.3

Thyroid cartilage	0.83	0.66 (0.30-0.80)	0.9	1.0	0.9	1.0	0.8	-	-

Glottic larynx	0.27	0.37 (0.11-0.81)	3.9	3.7	5.0	5.0	2.4	-	4.3

OAR = organ at risk, ICC = intraclass correlation coefficient, CI = concordance index and SD = standard deviation

### Concordance index

The mean CI values varied from 0.64 to 0.71, except for the glottic larynx for which the mean CI was 0.37 (Table 2). A large range in the CI of different observer pairs was seen (min-max, 0.11-0.86, Table 2).

### 3D analysis of variation

**Figure 3:**
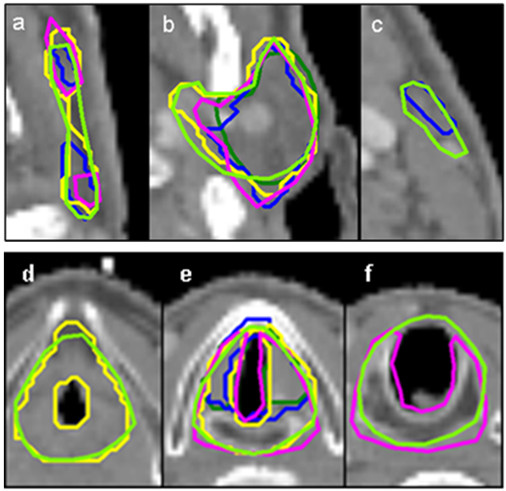
**Delineation variation of a parotid gland and a glottic larynx**. Left parotid gland and glottic larynx delineations in a typical cranial (**a **and **d**), central (**b **and **e**) and caudal (**c **and **f**) transverse CT slice. Each colour corresponds to one observer.

**Figure 4:**
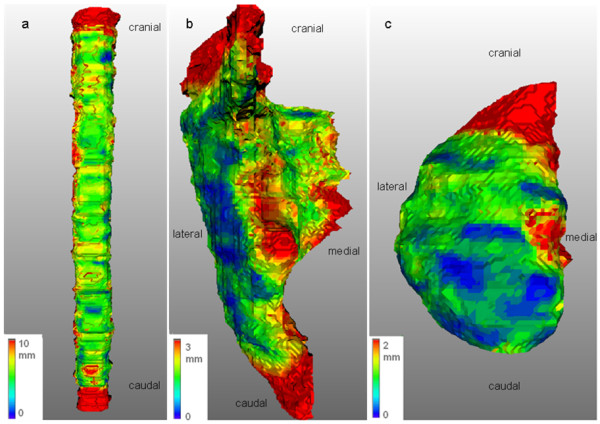
**3D delineation variation of a spinal cord, a right parotid and submandibular gland**. 3D Standard Deviations (SDs) for a typical patient plotted in colour scale on the median contour surface of the organ. Note the different scalings. Spinal cord (**a**), right parotid gland (**b**) and right submandibular gland (**c**), frontal view.

Largest interobserver variability in the 3D SD evaluation was found for the glottic larynx (global 3D SD of 3.9 mm, Table 2). Regional 3D SD values were up to 5.0 mm for the caudal and medial part of the glottic larynx. Figure 3(d,e,f) illustrates the variations in delineation of the glottic larynx for a typical patient CT. Best observer agreement was found for the thyroid cartilage (global 3D SD of 0.9 mm, Table 2). For all OARs, the regional 3D SD analysis showed largest variations in the cranial regions. Furthermore, medial regions tended to show more variation than lateral regions. Figure 4 illustrates the 3D regional variations of a typical patient for the spinal cord, and for a parotid and a submandibular gland. The predominating cranial, caudal and medial parotid gland variations can also be seen in Figure 3(a,b,c).

## Discussion

This study included an extensive 3D analysis of variation in delineation of a set of OARs in the head and neck area. All OARs, except from the glottic larynx, showed moderate interobserver variability with ICC values of 0.32-0.83, CI values of 0.64-0.71, and 3D SD values of 0.9-2.6 mm. Cranial, caudal, and medial regions of the OARs showed largest variations. The glottic larynx showed larger variation in delineation (ICC = 0.27, mean CI = 0.37 and 3D SD = 3.9 mm). All endpoints provided support for improvement of delineation practice.

The inaccurate results for consistency in delineation of the glottic larynx were mainly caused by poor compliance to the delineation guidelines. The guidelines prescribe the glottic larynx to end at the caudal edge of the cricoid, and to include the arythenoid cartilages in the glottic larynx contour. As illustrated in Figure 3(d,e,f), this description was not consistently followed. Reduction of the interobserver variability might be accomplished by joint delineation review sessions in which all radiation oncologists who are involved in head and neck cancer participate. These sessions are nowadays current practice at our institutes (UMCG, NCI-AVL).

The salivary glands showed moderate interobserver variability. Visual inspection of the parotid gland contours showed that the guidelines for these organs were also not consistently followed. The protocol prescribed that superficial temporal vessels should be enclosed in the delineated parotid gland, because they are generally hard to distinguish from the parotid gland tissue on scans with no or poor contrast. Still, some observers did not include the vessels in their delineation of the parotid gland. Joint delineation review sessions and enlightenment of the guidelines could help here. Yi and colleagues [18] for instance showed that clear stepwise delineation guidelines resulted in minimal variability. Our volume analysis of the parotid gland data showed similar CV values (12 and 15%) as data of Geets et al. [19] (17%). Nelms et al. [3] found larger CV values (34% and 29%), evaluated for 1 patient by 32 observers. Our 3D SD evaluation reflects valuable information on specific regional variations. Largest discrepancies for the parotid glands were located at the cranial, caudal and medial sub regions of the gland. For the submandibular glands, the cranial parts of the organ clearly showed largest discrepancies. Poor discrimination between tissues at the medial borders of the parotid gland (e.g. distinction from the posterior belly of the digastric muscle) and the cranial parts of the submandibular gland (e.g. distinction from the medial pterygoid muscle and the mylohyoid muscle) could be a reason for these larger 3D SD values. The addition of MRI might improve the visibility of borders between tissues [19,20]. The cranial and caudal variations for the parotid glands could partly be explained by the image resolution in the cranial-caudal direction of 2 mm (the slice thickness) and the fact that observers could only delineate on transverse CT slices, which limits the resolution in both the cranial and caudal part of the delineations. These limitations do, obviously, apply to the delineations of all OARs. Possibly the availability of delineation on multiple orientations could help to diminish these variations, as is also suggested by Steenbakkers et al. [17]. The use of a standardized delineation environment and tools for automatic contouring might further contribute to reduce interobserver variability [17,21,22].

Interobserver variability of the spinal cord was predominantly caused by variations at the cranial and caudal part of the structure, due to indistinctness of the guidelines and low compliance. This problem could be reduced by clearer delineation guidelines although it is unlikely that these variations will have major consequences in clinical practice as long as the spinal cord is accurately delineated in the vicinity of the irradiated volume and the maximum dose to the cord is considered as the leading parameter for treatment planning.

We analysed the interobserver variability in contouring on twelve CT sets, which consisted of six CT_plan _scans and six CT_rep _scans. The three endpoints of interobserver variability did not indicate a trend in the differences between CT_plan _and CT_rep _(for example see Figure 2). The correspondence of interobserver variability between the scans may suggest that the use of contrast (in CT_plan_) and the use of a(n) (observer specific) delineation template (in CT_rep_) have effects of comparable magnitude on the variation in delineation amongst observers, for the considered OARs. Besides, the guidelines are developed to be applicable to non-contrast as well as to contrast enhanced CT data, which will minimize possible variation in delineation due to (lack of) contrast. According to our experience the addition of contrast in delineating OARs is limited, because the uptake of contrast by the selected OARs is deniable.

We used different endpoints to quantify interobserver variability in head and neck OAR delineation. Variations in volume were indicated by the ICC and differences in combined volume and positional variations by the CI. Local variation in delineation was finally described by the *regional *3D SD. The results showed that the variation in the determination of the volume alone (ICC) can be rather large while the combined volume and positional variations (CI) did not point to such a large variability (e.g., the spinal cord showed ICC = 0.32, CI = 0.63). This implies that the variations are situated at the borders of the OAR rather than in positional mismatches of the centres of gravity. In another case the ICC indicated substantial agreement while the CI was relatively moderate (e.g., the thyroid cartilage showed ICC = 0.83 and CI = 0.66), which could indicate a substantial consistency in defining volume size while the centre of gravity of the volumes are shifted in position with respect to each other. So information of the ICC combined with the CI could help to identify the type of interobserver variation (in volume and position). To study variations between delineations in detail, the 3D SD provides most complete information.

Some of the endpoints to describe interobserver variability as used in the current study have also been applied in studies dealing with head and neck target volume interobserver variability. Geets et al. [19] found CVs of 4% and 20% for oropharyngeal and laryngeal-hypolaryngeal GTVs, which are more or less similar to the CVs we found for OARs (2-16%), excluding the glottic larynx (56%). Rasch et al. [20] described 3D SD variability for head and neck target volume delineation in the same range as our OAR results; 3.3-4.4 mm for the CTV and 4.9-5.9 mm for the elective nodal areas, while our global 3D SD results varied from 0.9 to 3.9 mm. Our results thus strengthen earlier findings (e.g. of Nelms et al. [3]) that interobserver variability is not only an important issue in the delineation of target volumes but also plays a role in the delineation of OARs.

## Conclusion

Cranial, caudal, and medial regions of the studied head and neck organs at risk showed largest interobserver variability, due to indistinctness of the delineation guidelines, the larger image resolution in the cranial-caudal direction, the limitation of delineation on transverse slices, and poor discrimination in contrast from adjacent tissues. Potential measures to reduce current redundant variability in delineation practice are: (1) guideline development, (2) joint delineation review sessions, and (3) application of multimodality imaging. Other aspects that could contribute to more consistency in delineation are a standardized delineation environment with standard delineation tools, the possibility to delineate on multiple orientations and automatic contouring tools. The latter should however carefully be validated using base line data of contouring variability such as the results of this study. Minor interobserver variability could ultimately benefit radiation oncology practice since it may contribute to more general applicability and improvement of TCP and NTCP models.

## Competing interests

The authors declare that they have no competing interests.

## Authors' contributions

CB carried out the design of the study, the analysis of the data, and drafting the manuscript. RS, AN, HB, OC and FB delineated the 12 CT sets. EvdH participated in the methodological design, statistical analysis of the data, and drafting of the manuscript. JD participated in the analysis of 3D variation in delineation. RS helped with the interpretation of the results and drafting of the manuscript. HM was the initiator of the study and helped to draft the manuscript. JL and AvV helped with critical revision of the manuscript. All authors read and approved the final manuscript.
